# Survey of PDA management in very low birth weight infants across Italy

**DOI:** 10.1186/s13052-020-0773-0

**Published:** 2020-02-14

**Authors:** Benjamim Ficial, Iuri Corsini, Stefano Fiocchi, Federico Schena, Irma Capolupo, Rosa Maria Cerbo, Manuela Condò, Daniela Doni, Simona La Placa, Salvatore Porzio, Katia Rossi, Sabrina Salvadori, Marilena Savoia, On the behalf of the Italian Study Group of Neonatal Cardiology of the Italian Society of Neonatology

**Affiliations:** 1 Neonatal Intensive Care Unit, Azienda Ospedaliera Universitaria Integrata di Verona, P.le Stefani 1, 37126 Verona, Italy; 2 Division of Neonatalogy, Careggi University Hospital of Florence, Florence, Italy; 3 Neonatologia e Terapia Intensiva Neonatale, Ospedale Valduce, Como, Italy; 4 Neonatal Intensive Care Unit, Fondazione IRCCS Cà Granda Ospedale Maggiore Policlinico di Milano, Milan, Italy; 5 Neonatal Intensive Care Unit, Ospedale Pediatrico Bambino Gesù, Rome, Italy; 6 Neonatal Intensive Care Unit, Fondazione IRCCS Policlinico San Matteo, Pavia, Italy; 7 Neonatal Intensive Care Unit, Ospedale A. Manzoni, Lecco, Italy; 8 Neonatal Intensive Care Unit, FMBBM San Gerardo, Monza, Italy; 9 Neonatal Intensive Care Unit, AOUP Giaccone, Palermo, Italy; 10 Neonatal Section, San Michele Hospital, Maddaloni, NA, Italy; 11 Neonatal Intensive Care Unit, Policlinico di Modena, Modena, Italy; 12 Neonatal Intensive Care Unit, Azienda Ospedaliera-Università di Padova, Padova, Italy; 13 Neonatal Intensive Care Unit, Azienda Ospedaliera Universitaria S Maria della Misericordia, Udine, Italy

**Keywords:** Survey, Preterm infants, PDA management, Neonatologist performed echocardiography

## Abstract

**Background:**

The optimal management of PDA in very low birth weight (VLBW) infants is still controversial. Aim of our study was to investigate the management of PDA in the Italian neonatal intensive care units (NICU).

**Methods:**

We conducted an on-line survey study from June to September 2017. A 50-items questionnaire was developed by the Italian Neonatal Cardiology Study Group and was sent to Italian NICUs.

**Results:**

The overall response rate was 72%. Diagnosis of PDA was done by neonatologists, cardiologists or both (62, 12 and 28% respectively). PDA significance was assessed by a comprehensive approach in all centers, although we found a heterogeneous combination of parameters and cut-offs used. None used prophylactic treatment. 19% of centers treated PDA in the first 24 h, 60% after the first 24 h, following screening echocardiography or clinical symptoms, 18% after the first 72 h and 2% after the first week. In the first course of treatment ibuprofen, indomethacin and paracetamol were used in 87, 6 and 7% of centers respectively. Median of surgical ligation was 3% (1–6%).

**Conclusions:**

Significant variations exist in the management of PDA in Italy. Conservative strategy and targeted treatment to infants older than 24 h with echocardiographic signs of hemodynamic significance seemed to be the most adopted approach.

## Backgrounds

Despite decades of research, there is still controversy on the optimal management of patent ductus arteriosus (PDA) in very low birth weight (VLBW) infants [1]. While in term infants PDA usually closes within 72 h of life, in preterm closure is delayed, allowing blood to flow from the aorta to the pulmonary arteries, leading to systemic under-perfusion and pulmonary over-circulation. The magnitude of the left to right shunt is responsible of the hemodynamic consequences of PDA and it is associated to various clinical problems: pulmonary hemorrhage, hypotension, intraventricular hemorrhage (IVH), bronchopulmonary dysplasia (BPD), necrotizing enterocolitis (NEC), mortality [2, 3].

In the past, to reduce the burden of these comorbidities, early routine closure of PDA was the preferred approach but did not improve long-term outcomes [4]. Fifty randomized controlled trials (RCTs) enrolling 4878 infants showed that early routine closure of PDA has no effect on the most important outcomes (BPD, NEC, neurodevelopmental impairment) with narrow confidence intervals [5].

In the last ten years, the lack of demonstrable improvement following non selective PDA treatment paved the way to a more conservative approach, that led to lower rates of medical and surgical closure, without compromising long-term outcomes [6].

On the other side, the increasing utilization of echocardiography by neonatologist, namely Neonatologist Performed Echocardiography (NPE), allowed targeting treatment to neonates with high/moderate ductal shunt, those were likely to benefit from PDA closure [7]. There is emerging evidence that prolonged exposure to high/moderate ductal shunt is associated with BPD and that early pharmacological treatment might reduce the incidence of BPD, although there is still no robust evidence from RCTs that treating these high-risk patients improves outcome [8, 9]

Currently, there is no clear evidence to help neonatologists identify which patient to treat, if any, when and with which drug, therefore the approach to both diagnosis and treatment of PDA has huge variations within individual centers, between institutions and countries [10]. Data on the current management of PDA were previously reported, but, to the best of our knowledge, no data were available from Italy [11–13].

Aim of our study was to investigate the management of PDA in VLBW infants across the Italian NICUs.

## Methods

We conducted a prospective cross-sectional survey study from June to September 2017.

### Questionnaire

Four members (I.C., B.F., S.F., F.S.) of the Italian Study Group of Neonatal Cardiology (ISGNC) developed a 50-item questionnaire according to the CHERRIES method for internet e-surveys [14]. The questionnaire was divided into 4 sections: diagnosis of a hemodynamically significant PDA (hsPDA), pharmacological, non-pharmacological and surgical management. All questions were loaded to the Google Forms Website, a free tool for creating online survey forms (https://docs.google.com/forms/u/0/) and were proofread. Prior to distribution, the survey was pilot-tested to identify potential inaccuracies by the other 9 members of the ISGNC and modified accordingly, as previously reported [15]. The questionnaire required approximately 30 min to be completed.

### Web-based survey

A cover letter containing a hyperlink to the survey was sent to the directors of the Italian Neonatal Units and to one attending neonatologist at each site with expertise in neonatal cardiology, based on the database of the Italian Society of Neonatology and the ISGNC. To ensure only one response for center an unique survey link was assigned to each institution. To prevent incomplete answers, the survey form could be submitted only when completed.

The first email was sent in June 2017 and a reminder in September 2017. No responders were subsequently contacted by a personalized e-mail and/or a phone call. No financial rewards were offered for taking part in the survey. Completion of the questionnaire implied consent to take part in the survey. The identity of each participant was kept confidential throughout the data collection and analysis. The missing responses of some centres defined the study as ‘voluntary inquiry with presence of non-respondents’.

### Data analysis

Google Forms automatically converted every questionnaire into Excel files (Microsoft, Seattle, WA). I.C. and B. F checked every questionnaire for possible inconsistencies throughout this process of conversion. Continuous variables were tested for normality using the Shapiro-Wilk test and presented as means (SD) or median (IQR) as appropriate. Categorical variables were presented as proportions. Comparisons between subgroups were conducted using a Student t test or a Mann-Whitney U test as appropriate. Categorical variables were compared using the χ^2^ or Fisher exact test as appropriate. A *p* value of < 0.05 was considered significant. SPSS v 20 (SPSS Inc., Chicago, Illinois) was used to perform the statistical analysis.

## Results

### Epidemiological and organisational characteristics of the centers

**Fig. 1:**
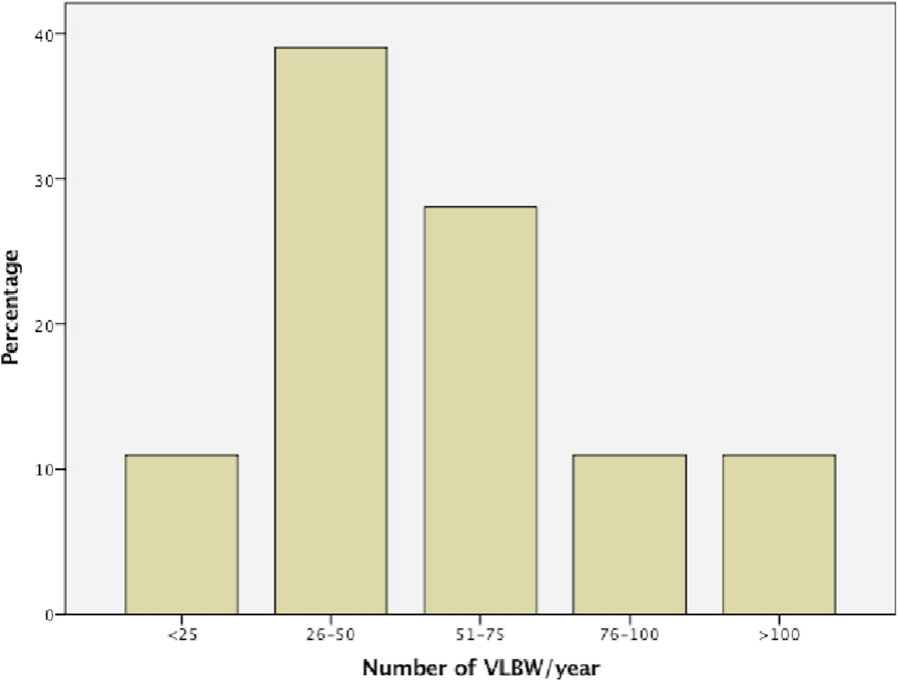
Italian NICUs divided according to the volume of activity, defined as the number of very low birth weight (VLBW) infants per year

The overall survey response rate was 72% (82/114). Among respondent centers, 85% were classified as level III and 15% as level IV neonatal units [16]. Comparing answers of early versus late respondents (i.e. after phone call reminder), we did not find statistically significant differences. In Fig. 1 the centers are divided according to the number of VLBW per year.

### Diagnosis of PDA

In 68% of centers there was an institutional protocol for PDA management. Neonatologist, cardiologist or both usually performed the echocardiography to assess a PDA in a VLBW neonate in 60, 12 and 28% of centers respectively. The first echocardiogram was performed in the first 24 h of life, between the 24 and 72 h or after the first 72 h in the 31, 60 and 9% of centers respectively.

In 87% of centers the first echocardiogram was always a complete structural examination to exclude the presence of CHD. In the remaining cases, a complete examination was performed only if CHD was suspected (7%) or before starting treatment (6%). Neonatologist, cardiologist or both excluded CHDs in the 51, 27 and 22% respectively.

**Fig. 2:**
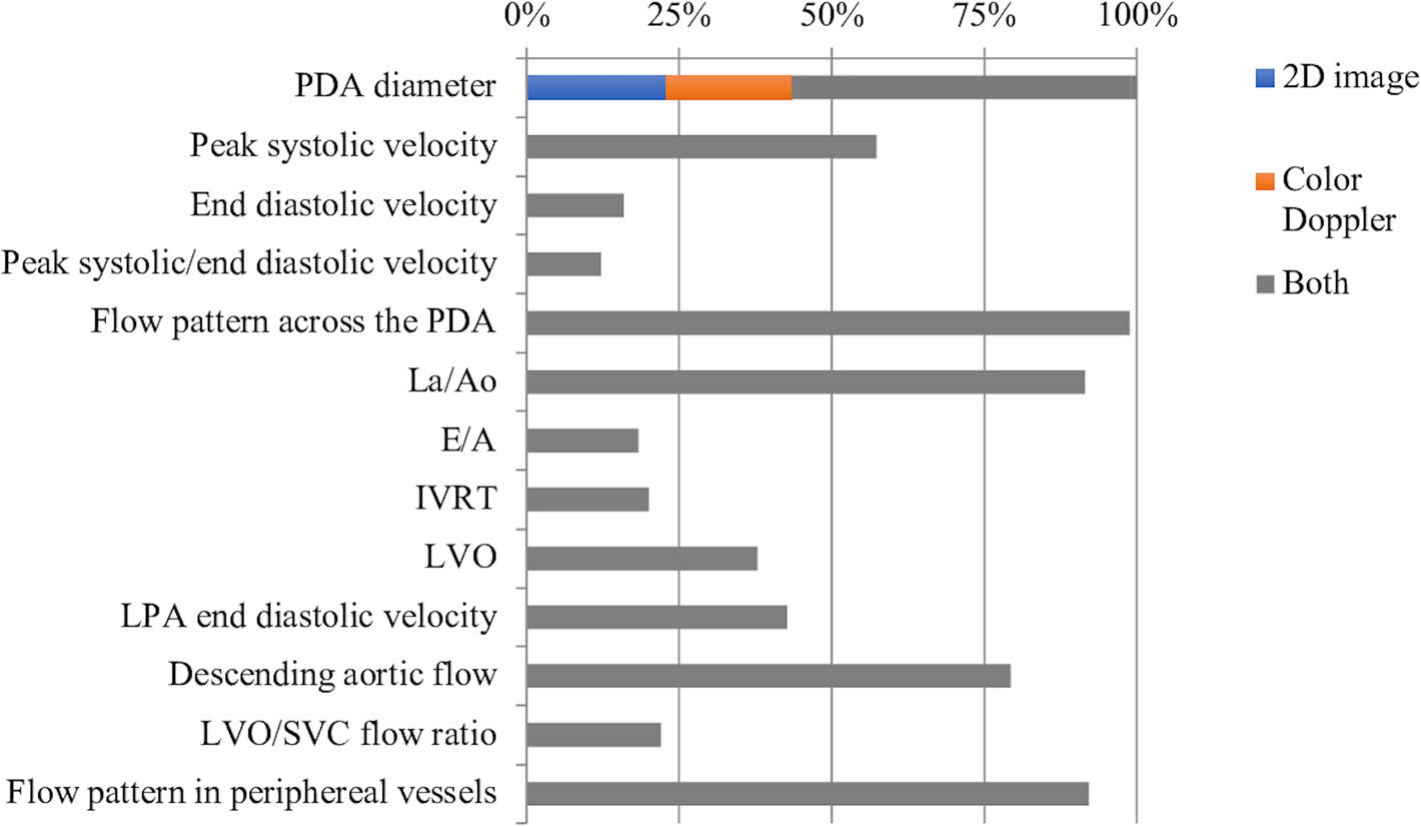
Echocardiographic parameters used among Italian NICUs for the assessment of PDA and their percentage of use. *LA* Left Atrium, *Ao* Aorta, *E* Mitral E wave, *A* Mitral A wave, *IVRT* Isovolumic relaxation time, *LVO* Left ventricular output, *LPA* Left pulmonary artery, *SVC* Superior vena cava

Figure 2 shows all the echocardiographic parameters used for the assessment of PDA and their percentage of use.

PDA diameter was measured at its narrowest point at the pulmonary end from a 2D image, from a color Doppler flow image or both in 24, 20 and 56% respectively.

In 74% of centers, the absolute value of PDA diameter was used. The PDA diameter was indexed to patient weight and to body surface area in 24 and 1% of centers respectively.

Median cut-off value for hsPDA diameter was 1.5 mm (1.5–2 mm) and for PDA diameter indexed to body weight was 1.4 mm/kg (1.4–1.5 mm/kg).

Flow across a PDA was assessed as follows: peak systolic flow velocity in the 57% of centers, end diastolic flow velocity in the 16%, ratio between peak systolic and end diastolic flow velocities in the 12%, type of flow pattern across a PDA in the 99%.

Pulmonary over-circulation was assessed as follows: left atrium/aortic root ratio (LA/Ao) in 95% of centers, ratio between E and A wave of transmitral flow (E/A) in 18%, left ventricular output (LVO) in 37%, end-diastolic flow velocity of left pulmonary artery (LPA) in 43%, left ventricle isovolumic relaxation time (IVRT) in 1%.

Among centers that used LA/Ao, median cut-off value of hsPDA was 1.5 (1.4–1.5).

Systemic hypo-perfusion was assessed as follows: descending aortic flow pattern in 79% of centers, flow pattern in peripheral vessels in 83%, LVO/SVC flow ratio in 22%.

The peripheral vessels examined were cerebral, celiac or mesenteric and renal in the 90, 69 and 31% respectively.

Calculation of resistance index, qualitative evaluation of flow pattern or both were used in the 31, 17 and 52% respectively. Table 1 compares the percentage of use of echocardiographic parameters by neonatologists and cardiologists.

**Table 1:** Comparison between echocardiographic parameters used by neonatologist and cardiologist in PDA assessment. *E* mitral E wave, *A* mitral A wave, *LA/Ao* Left Atrium Aortic root ratio, *IVRT* Isovolumic relaxation time, *LVO* Left ventricular output, *LPA* Left pulmonary artery, *SVC* superior vena cava

Echocardiographic parameter	Echo assessment by neonatologist	Echo assessment by cardiologist	p
PDA diameter	100%	100%	
Ductal Peak systolic velocity	54%	80%	
Ductal End diastolic velocity	14%	30%	
Ductal Peak systolic/End diastolic velocity	13%	10%	
Flow pattern across PDA	100%	90%	
LA/Ao	95%	100%	
E/A	19%	10%	
IVRT	1%	0%	
LVO	40%	20%	
LPA end diastolic velocity	44%	30%	
Descending aortic flow	79%	80%	
LVO/SVC flow ratio	22%	20%	
Flow pattern in peripheral vessels	89%	40%	*

* = ***p*** **< 0.05**

Only in 8% of centers the echocardiographic assessment of PDA satisfied the requirements proposed by the recently released indications of the “European Special Interest Group Neonatologist Performed Echocardiography” [17].

Near infrared spectroscopy (NIRS) was used in the 25% of centers: cerebral district alone was evaluated in the 6% of centers whereas both cerebral and renal district were evaluated in the 19%.

Most centers (84%) did not use biochemical markers when assessing a PDA, 13 and 3% of centers used brain natriuretic peptide (BNP) and troponin respectively.

### Medical treatment

Medical treatment of hsPDA was decided by pediatric cardiologist, neonatologist or neonatologist in collaboration with cardiologist in 3, 57 and 40% of centers respectively. Median incidence of medical treatment was 22% (17–30%).

**Fig. 3:**
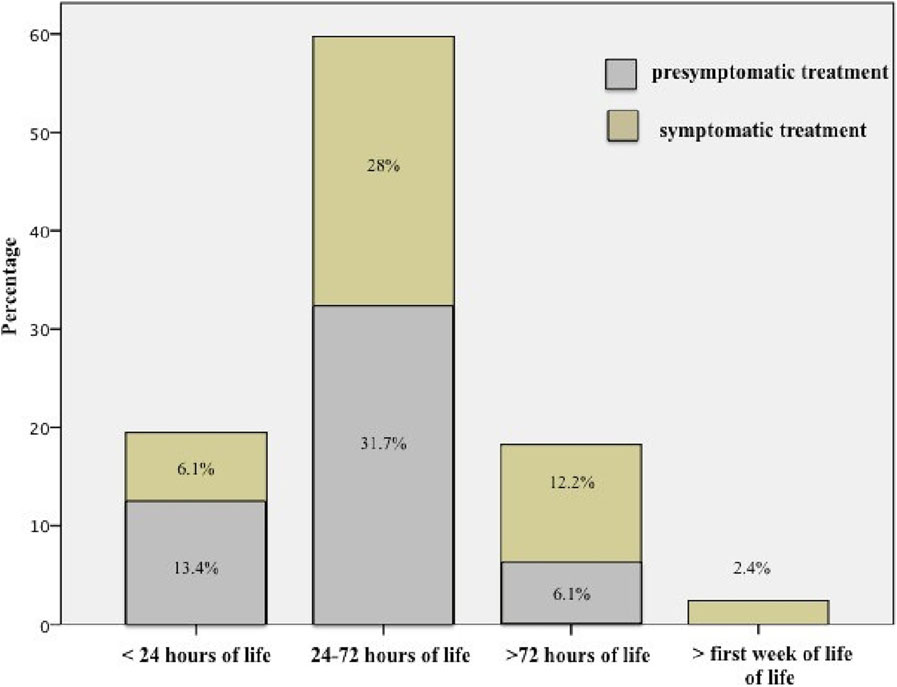
Timing (before 24 h of life, between 24 and 72 h of life, beyond 72 h of life, beyond the first week of life) and treatment strategy (presymptomatic/symptomatic) of pharmacological closure of PDA among Italian NICUs

Prophylactic treatment was not adopted among Italian NICUs. Figure 3 shows timing and treatment strategies of medical treatment of PDA among Italian NICUs. The timing of the first course of medical treatment showed marked variability. Nineteen per cent of centers started PDA treatment in the first day of life (13% based on pre-symptomatic echocardiographic screening, 6% based on signs and/or symptoms of PDA).

The majority of Italian NICUs did not treat babies in the first 24 h and considered pharmacological closure of PDA between the first 24 and 72 h of life (60%), adopting both a pre-symptomatic and symptomatic treatment (32 and 28% respectively). Eighteen percent of centers considered treatment of PDA only after the first 72 h of life (12% symptomatic and 6% pre-symptomatic) and 2% of centers did not treat PDA in the first week of life.

Comparing frequency of medical treatment in centers that adopted pre-symptomatic and symptomatic treatment we did not found significant differences (median 22% for both groups).

In the first course ibuprofen, indomethacin and paracetamol were used in 87, 6 and 7% of centers respectively.

Ibuprofen was administered in bolus, continuous infusion and orally in the 91, 5 and 4% respectively. The most common ibuprofen regimen was 10 mg/kg for the first dose followed by 5 mg/kg for the remaining two doses (92%). In 5% of centers ibuprofen 20 mg/kg for the first dose, followed by 10 mg/kg was used. In 2% both low and high dose ibuprofen regimens were used. The majority of the units (73%) used a 3 days course, instead 27% of units stopped the course earlier if PDA closed at daily follow-up echos.

Indomethacin was given by intravenous bolus infusion. In the majority of centers (95%) the first dose was 0.2 mg/kg and the following doses ranged from 0.1 to 0.25 mg/kg according to the day of life (0.1 mg in the first 48 h, 0.2 mg/kg from day 3rd to 7th of life, 0.25 mg/kg after the first week). The duration was three or six doses (57 and 3% respectively). Forty percent of centers stopped the course earlier if PDA closed.

Paracetamol was given orally, intravenously (bolus infusion) or both in the 68, 18 and 14% respectively. The most common dose regimen was 15 mg/kg four times a day (92%), followed by 10 mg/kg and 7.5 mg/kg four times a day (6 and 2% respectively). The duration of the cycle of paracetamol was 3 or 6 days in the 49 and 24% respectively. Twenty-seven percent of centers stopped the course earlier if PDA closed.

If the first course failed to close the duct, a second course with ibuprofen, indomethacin or paracetamol was used in 67, 14, 11% respectively. In 3% of centers patients underwent to surgical ligation and in the remaining 5% “watchful waiting”.

Among centers that used a second course of ibuprofen, the dose regimen was 10 mg/kg for the first dose and 5 mg/kg for two doses in the 74% of cases and 20 mg/kg for the first dose and 10 mg/kg for two doses in the 26%.

If the second course failed, the management options were: surgical ligation in 46%, a third course with ibuprofen (10%), indomethacin (23%) or paracetamol (15%), “watchful waiting” (6%).

The most frequent contraindications of ibuprofen and indomethacin were: diuresis < 0.5 m/kg/h (41%), diuresis < 1 ml/kg/h (47%), Platelets < 50.000/μL (77%), Platelets < 30.000/μL (17%), sepsis (30%), IVH ≥ grade 3 (90%), NEC (80%), increased creatinine (6%), every grade IVH (2%).

To treat PDA in babies with severe IVH or NEC, paracetamol, surgical ligation or conservative management followed by course with ibuprofen were adopted in 44, 33 and 23% of centers respectively.

### Supportive therapy

Fluid restriction was used in all infants with PDA in 47% of centers and only in infants with hsPDA in 43%. In 10% of centers fluid restriction was not used.

Enteral feeding was continued, reduced or totally withdrawn during pharmacological closure of PDA in 56, 37 and 7% of centers respectively.

Seventy per cent of centers did not used gastro-protective drugs during treatment with NSAIDs, 26 and 5% used ranitidine and omeprazole respectively.

Thirty-three per cent of centers used increased PEEP values for neonates with hsPDA.

Treatment of congestive heart failure varied between centers: standard dose furosemide (1–2 mg/kg) was used in 81% of centers, high dose furosemide (> 2 mg/kg) in 6%, fluid restriction in 67%, digoxin in 3%, dobutamine in 1%, hydrochlorothiazide and spironolactone in 6%.

### Surgical treatment

Surgical ligation was decided by pediatric cardiologist, neonatologist or neonatologist in collaboration with cardiologist in 7, 44 and 49% of centers respectively. Median incidence of surgical ligation was 3% (1–6%).

Surgical ligation was performed by cardiac surgeons of the same institution or of other institutions in 18 and 60% respectively. Surgical candidates were transferred to referral centers in the remaining 22%. In 91% of centers surgical ligation was performed at the cot-side in the NICU.

Surgical ligation was performed after failure of the first, second or third cycle of medical therapy in 3, 46 and 44% of centers respectively. In the remaining 7% of centers surgical ligation was not considered. Thirty-three per cent of centers performed surgical ligation immediately without trying medical therapy in babies with NEC or severe IVH. After surgery 79% of centers performed echocardiographic screening for post-ligation cardiac syndrome.

## Discussion

We investigated the PDA management in Italian neonatal units, the survey received a high response rate, that, although not optimal, was comparable to previous similar reports [18, 19]. Although the suboptimal response rate may be considered a limitation of the current survey, studies of non-response to pediatric surveys found that lower response rates were not necessarily related to bias [18]. Moreover, we found a low non-response bias comparing early versus late respondents. Therefore, we may conclude that our results were representative of the current clinical practice in Italy.

We found a great variability in diagnosis, medical, surgical and supportive treatment, as previously reported in other countries [ 11–13, 15]. Lack of evidence-based guidelines for the management of PDA was likely to be a major contributor to this variability [3]. Our survey was sent to a single neonatologist at each site with expertise in neonatal cardiology, we did not directly investigated intra-institutional practice variations. We speculate that major differences were likely to exist also within the same unit, only 68% of centers having institutional protocol for PDA management in place, similarly to other countries [10, 11, 15].

### Variability in approach to diagnosis

An ECHO-based approach for diagnosing ductal patency and assessing hemodynamic significance was widely adopted in Italian NICUs, according to the current evidence that proposes echocardiography as the most clinically applicable modality [1, 18]. The homogeneous implementation of an ECHO-based approach is likely to have different reasons. First, the vast majority of Italian NICUs had access to NPE, as shown from our previous survey: NPE was performed in 95% of centers and in 70% of centers on a 24/7 basis [20]. Secondly, clinical signs of PDA take 2 to 3 days to appear and have poor sensitivity and specificity [21]. Finally, early comprehensive echocardiography is able to assess shunt volume entity, possibly identifying neonates at higher risk of PDA comorbidities [22, 23].

Italian NICUs consistently used comprehensive echocardiography to assess shunt volume rather than an approach based on single parameter (i.e. ductal diameter), allowing for a more accurate evaluation of shunt entity. Reliance only on PDA size has been recently put in doubt, as the vast majority of RCTs that based interventions only on ductal diameter failed to show any improvements in PDA related morbidities [24] Many combinations of ultrasound parameters were proposed as surrogates of shunt volume to define a hsPDA, nevertheless robust agreement is proving difficult among neonatologists [25].

This was confirmed by our data: we found a heterogeneous combination of parameters meant to assess PDA. The combination more frequently adopted in Italy was similar to the one reported by a French survey [11]. No significant differences were found comparing cardiologist and neonatologist approach, except for the evaluation of flow reversal in peripheral vessels.

Following survey completion, a consensus statement of ESPR/ESN has been released aiming to standardize the echocardiographic assessment of hemodynamic significance of a PDA in terms of parameters used and cut-offs [17]. A minority of our centers met these essentials requirements, in particular, LVO, that is considered an essential index of pulmonary over-circulation, was adopted by a minority of centers and needs to be implemented.

Similarly to other European countries, Australia and New Zealand, echocardiographic assessment of PDA was more frequently performed by neonatologists rather than cardiologists [10–13].

In a minority of cases, the first evaluation was not a comprehensive study aimed to confirm normal cardiac anatomy, as recommended by the recently released ESPR/ESN consensus statement [26, 27]. Although in the remaining cases normal heart structure was confirmed afterwards, this is a major concern of the NPE assessment of PDA due to the risk of misdiagnosing critical CHD [26].

Considering the number of serial echo assessments required in preterm neonates, the need to integrate the ultrasound findings with clinical data and the limited access to pediatric cardiology service in Italy, echocardiographic assessment of PDA should probably remain the domain of a properly trained neonatologist, in collaboration with cardiologist whenever needed, to minimize the risk of misdiagnosis of critical CHDs [20]. In order to meet training needs and to pursue quality assurance and patient safety, our INCSG is currently designing and implementing a formalised and accredited training program in close collaboration with the Italian Society of Pediatric Cardiology (SICPED). This will be of major help to uniform and standardize the echocardiographic PDA assessment among neonatal units in Italy.

### Variability in approach to medical treatment

Approach to medical treatment of PDA varied markedly among Italian NICUs, yet we found an overall tendency towards a conservative management, in which pharmacotherapy and/or surgical ligation decreased, similarly to the data recently reported by other countries (USA, Canada) [28, 29]. Data reported by the Italian Neonatal Network (Vermont Oxford Network) for the year 2017 confirmed our results, despite Survey method was not the optimal tool to assess the incidence of medical treatment of PDA among Italian NICUs.

Early medical treatment between 24 and 72 h of life seemed to be the most adopted strategy. This approach is supported by recent data showing that treating babies with high to moderate shunt volume across the PDA in the first 2–3 days of life might reduce BPD. Apparently, a critical window may be present during the first seven to ten days of life, in which exposure to a high-moderate shunt through PDA may increase the risk of BPD and pulmonary comorbidities. After this period, PDA treatment does not seem to influence BPD development [9]. The majority of Italian centers adopted a pre-symptomatic approach, performing early screening echocardiography before the onset of PDA clinical signs. This approach is based on the rationale that treating neonates at a preclinical stage might avoid PDA comorbidities. Short and long-term benefits of this strategy remain to be proven, despite some emerging data would show less pulmonary hemorrhage, in-hospital mortality and BPD compared to controls [7, 30]. Pre-symptomatic approach was less common in other European countries (UK and France), compared to Italy [11, 13]. These data were retrieved some years ago, recent advancements in NPE for PDA assessment may have led neonatologists to a change from previous practice [21].

Prophylactic indomethacin was not used: similarly to other countries, this approach, that gained popularity in the ‘90s, is declining, possibly because subsequent data did not find long-term improvements [4].

In the vast majority of centers ibuprofen was the drug of choice in the first course of medical therapy, whereas a minority of centers used indomethacin and paracetamol. Use of ibuprofen as first line drug was consistent with other European countries, considering that it carries decreased risk of renal impairment and similar efficacy [31].

Standard dosage regimen of ibuprofen was universally adopted by almost all Italian NICUs [32]. A minority of centers used the high dose regimen, in particular when the first course failed to close the duct [33].

Indomethacin use increased after first course of ibuprofen failed, possibly because indomethacin effectiveness is not affected by late treatment [34]. Short course and low dosing regimen were more commonly used, most likely because equally effective in ductal closure and inducing less renal impairment [35, 36].

We found a significant use of paracetamol, both as first line and as rescue therapy. There is mounting evidence of its efficacy, particularly where NSAIDs are contraindicated or unsuccessful [37].

Severe IVH and NEC were common contraindications to NSAIDs, as well as oliguria and thrombocytopenia, with yet different cut-offs.

### Variability in approach to supportive treatment

Despite conflicting evidence, conservative interventions have been increasingly adopted as a therapeutic modality in recent years [38, 39]. The lack of standardization of this practice was confirmed by our data: no standard criteria and modalities for fluid restriction, diuretics and end-expiratory pressure were adopted among Italian centers.

### Variability in approach to surgical ligation

Due to concerns about adverse neonatal outcomes, in the last decade surgical ligation rate decreased. Our data confirmed this tendency in Italian NICUs. In the vast majority of centers surgical ligation was performed at the NICU bedside by an on-site or an off-site team, to eliminate the risks associated with transferring instable babies to referring hospitals, according to the recent evidence [40].

## Conclusions

Approach to both diagnosis and treatment of PDA among Italian NICUs was highly variable and it would be interesting to confirm this variability in the management of PDA in a European perspective. No homogeneous criteria for PDA diagnosis were found, although an ECHO-based approach with a comprehensive echocardiographic assessment was widely adopted.

Conservative strategy and targeted treatment to infants older than 24 h with echocardiographic signs of moderate to high left to right shunt volume across the PDA seemed to be the most adopted approach. While awaiting further evidence on PDA management from ongoing trials, our data confirmed the need of standardization of clinical practice, which would hopefully improve clinical management and trials methodology and results.

## Supplementary information

**Additional file 1.** List of Neonatal Units respondent to the survey.

## Data Availability

The data that support the findings of this study are available from Italian Society of Neonatology but restrictions apply to the availability of these data, which were used under license for the current study, and so are not publicly available. Data are however available from the corresponding author upon reasonable request and with permission of Italian Society of Neonatology.

## Abbreviations

A: Mitral A wave
BNP: Brain natriuretic peptide
BPD: Bronchopulmonary dysplasia
CHD: Congenital heart disease
E: Mitral E wave
ESN: European Society of Neonatology
ESPR: European Society of Pediatric Research
hsPDA: Hemodynamically significant PDA
IQR: Interquartile range
ISGNC: Italian Study Group of Neonatal Cardiology
IVH: Intraventricular hemorrhage
IVRT: Isovolumic relaxation time
LA/Ao: left atrium/aortic root ratio
LPA: Left pulmonary artery
LVO: Left ventricular output
NEC: Necrotizing enterocolitis
NICU: Neonatal intensive care unit
NIRS: Near infrared spectroscopy
NPE: Neonatologist performed echocardiography
NSAID: Nonsteroidal anti-inflammatory drug
PDA: Patent ductus arteriosus
RCT: Randomized controlled trial
SD: Standard deviation
ISPC: Italian Society of Pediatric Cardiology
SVC: Superior vena cava
VLBW: Very low birth weight
