# Supplementary material for: Survey of PDA management in very low birth weight infants across Italy
Source: Ital J Pediatr. 2020 Feb 14;46:22. doi: 10.1186/s13052-020-0773-0 (PMC7023762; doi:10.1186/s13052-020-0773-0)
Supplement: Supplementary file 1 — Additional file 1. List of Neonatal Units respondent to the survey. [file 13052_2020_773_MOESM1_ESM.docx]

**List of Neonatal Units respondent to the survey**

Ospedale San Giovanni di Dio ASP, Agrigento; AON SS Antonio e Biagio e Cesare Arrigo, Alessandria; AOU Ospedali Riuniti, Ancona; Ospedale San Donato, Arezzo; Ospedale Di Venere, Bari; AO G. Rummo, Benevento; Ospedale Papa Giovanni XXIII, Bergamo; AOU Policlinico Sant’Orsola-Malpighi, Bologna; Ospedale Maggiore AUSL di Bologna; Ospedale di Bolzano; ASST Spedali Civili, Brescia; Ospedale A. Perrino, Brindisi; AOU Cagliari; Ospedale Cardarelli, Campobasso; AO Cannizzaro, Catania; AOU Policlinico Vittorio Emanuele PO G. Rodolico, Catania; PO Garibaldi-Nesima, Catania; Ospedale M Bufalini, Cesena; Ospedale Sant’Anna ASST Lariana, Como; Ospedale Valduce, Como; AO dell’Annunziata, Cosenza; Istituti Ospitalieri Cremona; Ospedale San Giovanni di Dio ASP Crotone; Umberto I, Enna; Azienda Ospedaliero-Universitaria di Ferrara; AOU Careggi Firenze; AOU Meyer, Firenze; Ospedale S Giovanni di Dio, Firenze; AOU Ospedali Riuniti di Foggia; Ospedale Vittorio Emmanuele, Gela; Ospedale San Salvatore, L'Aquila; P.O. "Vito Fazzi", Lecce; Ospedale A. Manzoni, Lecco; Ospedale Versilia, Lucca; AO Carlo Poma, ASST, Mantova; AOU Policlinico "G. Martino", Messina; Ospedale Barone Romeo di Patti, Messina; ASST Fatebenefratelli Sacco, Macedonio Melloni, Milano; ASST Rhodense, Milano; Fondazione IRCCS Cà Granda Ospedale Maggiore Policlinico, Milano; Grande Ospedale Metropolitano Niguarda, Milano; Policlinico di Modena; Ospedale San Gerardo, Monza; A.O dei Colli, Ospedale Monaldi, Napoli; Ospedale Evangelico Betania, Napoli; AORN A. Cardarelli, Napoli; Azienda Ospedaliero Universitaria “Maggiore della Carità”, Novara; Ospedale San Francesco, Nuoro; Azienda Ospedaliera-Università di Padova; PO Camposampiero, Padova; AO ARNAS Civico – Di Cristina – Benfratelli Palermo; AOU Policlinico Paolo Giaccone, Palermo; Ospedale Buccheri La Ferla, Palermo; Ospedale Ingrassia, Palermo; Ospedali Riuniti Villa Sofia-Cervello, Palermo; Azienda Ospedaliero Universitaria di Parma; Fondazione IRCCS Policlinico San Matteo, Pavia; AO Perugia; AOU Pisa; Ospedale San Carlo, Potenza; Nuovo Ospedale di Prato; ASP Ragusa; Grande Ospedale Metropolitano Bianchi Melacrino Morelli, Reggio Calabria; Arcispedale Santa Maria Nuova IRCCS, Reggio Emilia; AO San Camillo Forlanini, Roma; Fatebenefratelli-Isola Tiberina, Roma; Ospedale Pediatrico Bambino Gesù, Roma; Ospedale San Pietro FBF, Roma; Policlinico Gemelli, Roma; AOU S. Giovanni di Dio e Ruggi d’Aragona, Salerno; AOU Siena; Ospedale SS Annunziata, Taranto; AOU. Citta della Salute e della Scienza di Torino; Ospedale Maria Vittoria, Torino; ASP 9, Trapani; Ospedale Santa Chiara, Trento; Ospedale Cà Foncello, Treviso; IRCSS Burlo Garofolo, Trieste; Azienda Ospedaliera Universitaria S Maria della Misericordia, Udine; ASST Sette Laghi, Varese; AOUI Verona; Ospedale S Bortolo, ULSS8 Berica, Vicenza
